# Study on Shear Resistance of Aluminum Alloy Joints Enhanced by Surface Geometry

**DOI:** 10.3390/ma18091954

**Published:** 2025-04-25

**Authors:** Xiangke Zheng, Ning Hu, Linsen Shu, Xin Fu, Yuqi Wang, Dacheng Zhang

**Affiliations:** 1 School of Optoelectronic Engineering, Xidian University, Xi’an 710126, China; 2 Xi’an Institute of Optics and Fine Mechanics, Chinese Academy of Sciences, Xi’an 710119, China; huning826@163.com (N.H.); fuxing@opt.ac.cn (X.F.); 3 School of Mechanical Engineering, Shaanxi University of Technology, Hanzhong 723001, China; shulinsen19@163.com (L.S.); wangyuqi1117@126.com (Y.W.)

**Keywords:** surface micropattern, shear strength, adhesive joints, failure mode

## Abstract

To improve the shear strength of the 2A12 aluminum alloy adhesive-bonded joint, two kinds of surface micropatterns, parallel and cross waves, were constructed on the surface of aluminum alloy by a laser engraving machine. The shear strength of two different surface micropatterns at different laser processing distances was investigated. The results show that the surface of the aluminum alloy with a surface micropattern shows excellent hydrophilicity, which is beneficial to forming a mechanical interlock between the adhesive and aluminum alloy. The shear strength of the bonded joint decreases with the increase in laser processing distances for the parallel wavy micropattern. When the laser processing distance is 0.5 mm, the shear strength reaches a maximum of 14.04 MPa. For the cross-wave micropattern, the shear strength of the bonded joint increases first and then decreases with the increase in laser processing distances. When the laser processing distance is 0.75 mm, the shear strength reaches a maximum of 13.74 MPa. The obtained data are important for adhesive aluminum alloys with different surface micropatterns.

## 1. Introduction

In recent years, the adhesive bonding technology of aluminum alloy has been important to achieve lightweight structural materials and is widely used in the aerospace field [1,2]. In space cameras, the supporting material of the lens is usually aluminum alloy, in order to ensure the accuracy and stability of the mirror position [3]. Reliable adhesive bonding technology is required between the aluminum alloy and the lens. Adhesive bonding technology has the advantages of strong flexibility, low cost, and uniform stress distribution [4,5]. In the traditional connection mode, not only the rivet head will increase the joint weight but also stress concentration around the rivet hole can easily occur [6]. Welding can also realize the connection, but the heat generated in the welding process has a large influence on the mechanical properties of the joint [7]. Recent experimental investigations reveal adhesive joints outperform traditional methods and highlight their technological significance [8,9].

The bonding quality needs to be considered to improve the bonding strength. The bonding quality is affected by many factors, such as the surface treatment of the substrate before bonding, the thickness of the adhesive layer, and the curing conditions of the bonding [10,11,12]. When determining the properties of the substrate and the type of adhesive, its quality depends largely on the surface treatment of the substrate. However, cleaning the surface alone does not significantly improve the quality of the joint [13,14]. Chemical, mechanical, and physical methods are used to pretreat the substrate, which can significantly improve the bonding quality [15,16]. To improve the bonding strength of the adhesive, the methods used include increasing the adhesive area, changing the surface wettability of the substrate, and mechanical locking between the colloid and the substrate [17,18]. The shear strength of 7075 aluminum alloy before chemical corrosion is 7.64 MPa, and the shear strength after chemical corrosion can reach 12.96 MPa, which is 69.63% higher than that of untreated [19]. The shear strength of the aluminum joint is 19.3 MPa before grinding, and the maximum shear strength after grinding is 30.4 MPa, which is 57.5% higher than that of the unground substrate [20], both attributed to increased surface roughness. Although the use of chemical reagents and grinding methods has significant improvements in bonding strength, the treatment of chemical reagents has a better impact on the environment. Accordingly, the grinding method requires more time and is less efficient, making it difficult to carry out industrial mass production.

Laser pretreatment of the substrate surface is a non-contact surface quality processing method. The method can accurately control the energy and position of the laser beam and improve the processing accuracy, but also realizes the advantages of fast processing speed, saving processing time and processing cost, fast cleaning speed, and environmental protection [21,22]. Jiang et al. [23] designed different micropatterns (+ texture, × texture, and ❊ texture) on the surface of 6061 aluminum alloy by laser. The tensile shear strength of the treated joint can reach 24 MPa, which is 1225% higher than that of the untreated specimen. Voswinkel et al. [24] obtained a linear micropattern on substrates that reduced processing time by 66% while retaining 95% shear strength of untreated joints. Feng et al. [25] used a nanosecond laser to change the surface morphology of carbon fiber composites and found that the surface roughness and wettability of the treated substrates changed significantly. The results show that, when the laser power is 24.5 W, the maximum joint strength is 14.3 MPa, which is about 2.2 times that of the laser unengraved joint. Based on the above research, + texture, × texture, and ❊ texture and linear micropattern on substrates surface can improve the bonding strength. Among the same bonding materials and joint forms, as well as laser processing parameters, the main factors affecting the bonding strength are laser processing distances and micropattern type. The laser processing distances directly affect the density of micropattern, thereby affecting the contact area of adhesives. Therefore, this work mainly considers the influence of laser processing distances on the bonding strength of aluminum alloy joints with two micropatterns (parallel and cross waves).

Epoxy adhesives are widely used in the aerospace field, especially in the support structure of mirror bodies in space cameras, but the intermolecular force between epoxy adhesives and untreated substrate surfaces is relatively weak [26]. Therefore, the mechanical properties of the joint can be improved by fabricating micropatterns on the substrate surface. In the reported literature, the shear strength of the vertical pattern is lower than that of the 45° pattern and the 45°× pattern, where all pattern directions are based on the direction of action of the shear stress along the reference axis [27]. In this paper, parallel wave (PW) and cross wave (CW) micropatterns with different laser processing distances were engraved on a 2A12 aluminum alloy substrate by a laser engraving machine and compared with the bonding sample before engraving. In this process, firstly, the surface morphology, micropattern, and wettability of the substrate were characterized. Then, the adhesive interface was further analyzed by scanning electron microscopy, and a shear test was used to measure the shear resistance of the adhesive joint. Finally, the fracture morphology and fracture form were analyzed. This work expects to provide an important reference value for aluminum alloy bonded joints with different surface micropatterns.

## 2. Experimental Method

### 2.1. Materials

2A12 aluminum alloy (China Aluminum Corporation, Beijing, China) was selected as the substrate to be bonded and a butt joint was manufactured. The specifications are 10 mm × 10 mm × 5 mm, and the chemical composition of the substrate is shown in Table 1. The adhesive is 3M Scotch-Weld^TM^ 2216 epoxy adhesive (Shanghai, China) commonly used in optical machine construction. This adhesive consists of two components, namely components A and B, and is mixed according to the ratio of A:B = 7:5. Acetone is used as a surface cleaner to wipe all substrate surfaces.

### 2.2. Laser Surface Pretreatment

**Figure 1:**
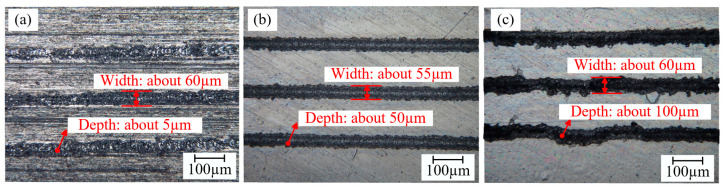
Surface morphology of Al alloy under different laser powers (**a**) 200 W; (**b**) 250 W; (**c**) 300 W.

**Figure 2:**
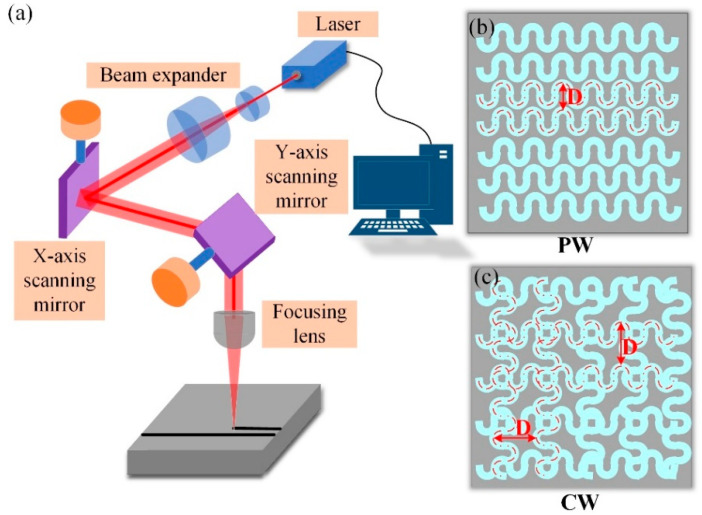
Laser surface treatment process (**a**) laser equipment working principle; (**b**) parallel wave micropattern; (**c**) cross wave micropattern.

In order to ensure that the surface of the aluminum alloy is free of contaminants and grease to better absorb the laser energy, the surface of the substrate is cleaned with acetone before the laser treatment of the aluminum alloy surface and allowed to dry completely before use. A laser engraving machine (LR-FIB-50T, Xi’an Langrui Laser Technology Corporation, Xi’an, China) was used to engrave parallel wave (PW) and cross wave (CW) micropatterns with different laser processing distances on the substrate surface. The maximum power of the laser engraving machine was 500 W. In order to ensure the clean shape of the texture and the appropriate depth of the groove, a large number of preliminary verifications of the parameters used in laser processing were carried out. In the preliminary validation experiment, the processing speed was set to 50 mm/s according to reference [28]; and the laser power was set to 200 W, 250 W, and 300 W, with a processing frequency of 3 times, as shown in Figure 1. An optical microscope (VHX-7000, Keyence Corporation of America, Elmwood Park, NJ, USA) is used to obtain the surface topography and groove dimensions under different laser processing powers. When the laser power is 200 W, the depth of the groove is about 5 µm, which is not conducive to the connection between the adhesive and the aluminum alloy, as shown in Figure 1a; when the laser power is 300 W, the depth of the groove is about 100 µm, and the aluminum oxide deposits appear at the edge of the groove, as shown in Figure 1c; when the laser power is 250 W, the depth of the groove is about 50 µm, and there is less alumina accumulation, as shown in Figure 1b. Therefore, in this study, experiments were conducted with different laser processing distances using a laser power of 250 W, a scanning speed of 50 mm/s, and three scanning times. To reduce experimental workload and reduce economic costs, according to the experimental results in Figure 1, better experimental parameters can be obtained. Therefore, the orthogonal experiments with three-factor parameters were not conducted. Figure 2 shows how the laser device works and the schematics of different micropatterns. Detailed laser processing parameters are given in Table 2.

### 2.3. Surface Analysis

All samples were first cleaned with acetone and then naturally dried. A contact angle measuring instrument (JC2000CS, Powereach, Shanghai, China) was used to measure the contact angle of distilled water on the surface of the laser-engraved substrate and unengraved substrate. A fixed volume method was used to measure the contact angle and a uniform drop volume of 3 µL was selected to assess how the wettability of the bonding area was affected before and after laser engraving.

### 2.4. Joint Fabrication and Interface Characteristics

Depending on the actual application, the method of making the butt joint is adopted. The thickness of the adhesive layer is typically 0.2 mm, and the thickness of the adhesive layer is controlled by a 0.2 mm wire. After bonding, the adhesive layer is fully cured at room temperature. After curing, to explore the bonding condition of the bonding interface, the bonding surface of the bonding joint after bonding is polished and polished to ensure that the bonding surface is smooth and clean. Then, scanning electron microscopy (JSM-7610FPlus, JEOL, Tokyo, Japan) and EDS were used to analyze the interfacial adhesion quality. In addition, since epoxy resin is non-conductive, a thin layer of gold is sprayed on the surface of the sample to provide electrical conductivity before observation using scanning electron microscopy.

### 2.5. Shear Test

**Figure 3:**
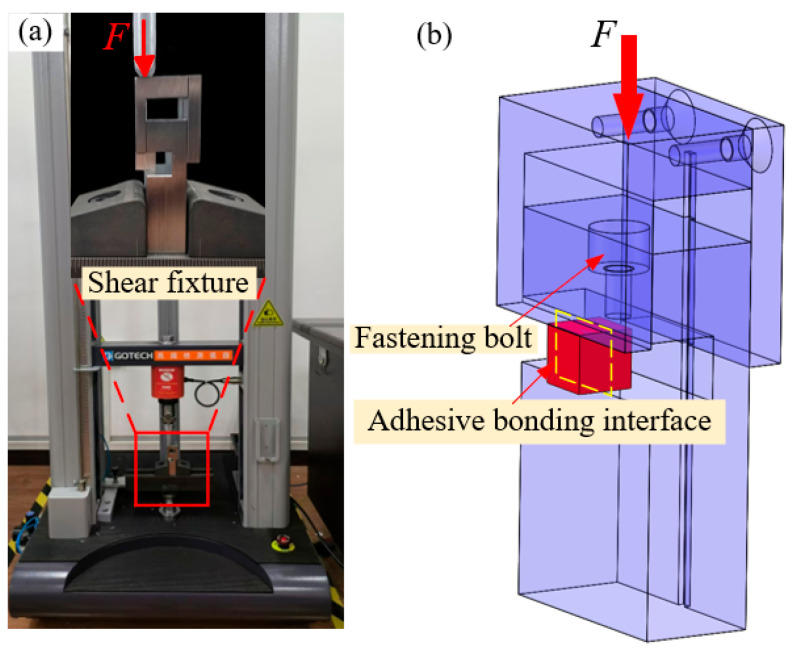
Shear performance test of bonded joints (**a**) shear test; (**b**) shear fixture diagram.

To determine the shear strength value of the aluminum alloy bonding joint, the test fixture was designed, as shown in Figure 3. One side of the adhesive specimen is fixed on the shear fixture by fastening bolts, and the other side is moved downward by the upper press block so that the adhesive joint is subjected to a pure shear stress state. A tensile testing machine (A1-7000-MU2, Gotech Testing Machines Inc., Taichung, Taiwan) was used for testing at a loading speed of 5 mm/min. To save the cost of material application and simplify the test, two samples were prepared for each group of parameters. An optical microscope system (VHX-7000) was used to observe the macroscopic morphology of the damaged specimens, and some microscopic regions of the damaged specimens were analyzed by scanning electron microscope.

## 3. Results and Discussion

### 3.1. Wettability Analysis

**Figure 4:**
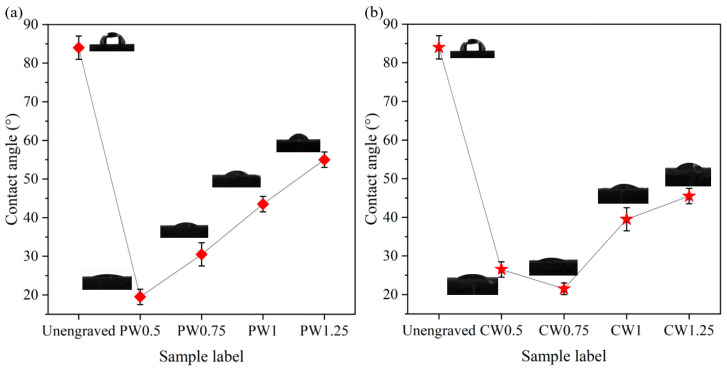
Contact angle of Al alloy surface (**a**) between the unengraved substrate and substrate surface with parallel wave shape, and (**b**) between the unengraved substrate and substrate surface with a cross wave shape.

The free energy of the substrate can be indirectly evaluated by measuring the contact angle of distilled water on the surface of the substrate. A smaller contact angle means higher free energy on the surface of the substrate, better wettability, and better adhesive bonding [23]. Figure 4 shows the contact angles of the substrate surface on the aluminum alloy. Before laser processing, the contact angle was approximately 84°. Figure 4a shows the surface contact angles of the unengraved substrate and substrate with the parallel wavy shapes. Compared to the unengraved substrate, the samples engraved with the laser have better wettability. When the laser processing distance is 0.5 mm, the sample numbered PW0.5 has the smallest contact angle of 19.5°, indicating good wettability at this distance. As the laser processing distance increases, the surface contact angle of the substrate gradually increases. The reason is that as the laser processing distance increases, the number of grooves decreases, which is not conducive to the spread of liquid on the substrate surface. Figure 4b shows the surface contact angle between the unengraved substrate and the substrate with cross-wave shapes; after laser processing, the sample exhibited good wettability. As the laser processing distance increases, the contact angle first decreases and then increases. This is because when laser etching the surface of the Al alloy, a part of the Al alloy substrate is melted, and after cooling, a recast layer is formed and deposited on both sides of the groove. A too-small laser processing distance increases the area of the recast layer, causing an obstruction to the flow and spreading of liquid on the substrate surface, thereby increasing the contact angle. In the samples CW1 and CW1.25, as the laser processing distance increases, a few grooves increase the contact angle of the substrate surface.

### 3.2. Shear Strength Analysis

**Figure 5:**
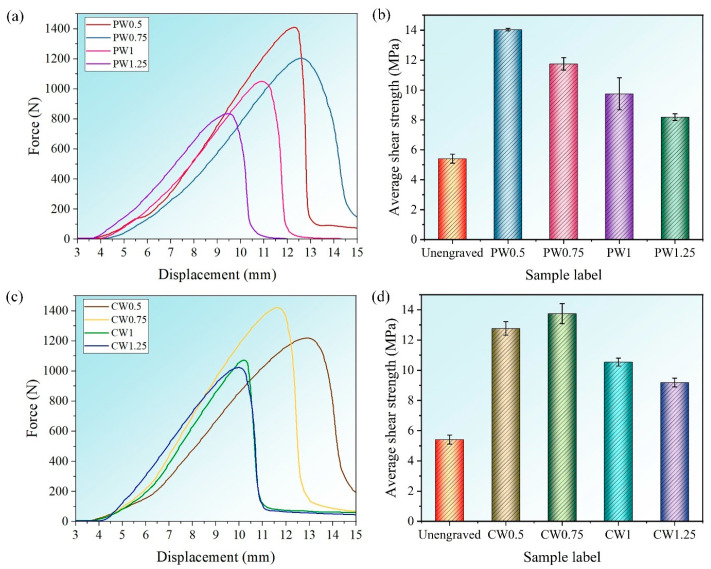
Load–displacement curve and shear strength of joint (**a**) load–displacement curves of joints with parallel wavy micropattern; (**b**) shear strength for joints with parallel wavy micropattern; (**c**) load–displacement curves of joints with cross wavy micropattern; (**d**) shear strength for joints with cross wavy micropattern.

Table 3 shows the measured values of shear strengths for each sample and average values. Figure 5 shows the shear strength of different adhesive-bonded joints. The shear strength of the bonded joint after laser engraving is much higher than that of the joint before engraving. The shear strength of the bonded joint without laser engraving is only 5.41 MPa. Compared with the unengraved sample, the joint shear strengths of the PW0.5, PW0.75, PW1, and PW1.25 samples after laser processing were increased by 159.5%, 117.2%, 80.2%, and 51.4%, respectively. The shear strength of the parallel wave and cross wave after laser engraving is higher than that of the bonded joint without laser engraving. It is considered that the laser processing distances have a certain effect on the bonding properties. Therefore, we need to discuss the effect of the wavy micropattern on the shear strength of aluminum alloy joints with different laser processing distances at the same power and processing speed. Figure 5a,c shows the representative load–displacement curves under different laser processing distances. Figure 5b,d shows the corresponding maximum average shear strength. For the parallel wavy micropattern, the shear strength of bonded joints decreases with the increase in laser processing distance. Among them, for the sample PW0.5, the shear strength reaches a maximum value of 14.04 MPa. The shear strengths of the PW0.75, PW1, and PW1.25 samples are 11.75 MPa, 9.75 MPa, and 8.19 MPa, respectively. Compared with PW0.5, the shear strength decreased by 16.31%, 30.56%, and 41.67%, respectively. This is because the engraving area gradually decreases with the increase in laser processing distance, thus reducing the bonding strength. For the cross-wave micropattern, the shear strength increases first and then decreases with the increase in scanning spacing. The CW0.75 sample has the highest shear strength (13.74 MPa). Compared with the unengraved sample, the joint shear strengths of the CW0.5, CW0.75, CW1, and CW1.25 samples after laser processing were increased by 136%, 154%, 94.8%, and 69.9%, respectively. For the sample CW0.5, because the laser processing distance is too close, the shear strength is low, reaching 12.77 MPa, which is reduced by 7.1% compared to CW0.75. The reason is that the laser processing distance is too close, and the residue in the trench will be deposited on the edge of the trench; this further reduces the effective width of the groove, causing the adhesive to not fully bond to the substrate, resulting in a decrease in bond strength. In the samples CW1 and CW1.25, the shear strengths reached 10.54 MPa and 9.19 MPa. Compared with CW0.75, they were reduced to 23.29% and 33.11%, respectively. This is because the laser processing distance is too wide, which reduces the adhesion area of the adhesive on the substrate. Therefore, suitable laser processing distances and surface micropatterns can effectively improve the bonding strength of the adhesive interface.

Based on the above analysis, the prepared wavy micropattern can increase the bonding strength by up to 159% compared to the unengraved sample. Compared with previous studies, when manufacturing linear micropatterns on aluminum alloys, the adhesive strength increased by 25% compared to unengraved samples [29]. Designing a pit pattern on the surface of aluminum alloy increases the shear strength by approximately 28.15%, compared to the original milled sample [30]. It is worth noting that in other literature, the use of the X-texture design has been reported to increase its shear strength by 1225% [23]. This is mainly due to the differences in bonding area, variations in adhesive types, and characteristics of the laser processing equipment, resulting in different adhesive performances. Therefore, the use of wavy micropattern has good engineering application value and academic research value in improving interfacial bonding strength.

The effects of different geometric parameters (laser processing distance: 0.5 mm, 0.75 mm, 1 mm, 1.25 mm) on the shear strength were analyzed by using the ANOVA method. Two samples were used in each group. A one-way ANOVA was performed, as shown in Figure 5b, and the results are shown in Table 4. Since the *F*-value (30.25) is greater than the critical value *F*_crit_ (4.2565) and the *p*-value is less than the threshold of 0.05, the laser processing distance has a significant effect on the shear strength of the parallel wavy joints. A one-way ANOVA was performed, as shown in Figure 5d, and the results are shown in Table 5. Since the *F*-value (51.786) is greater than the critical value *F*_crit_ (4.2565) and the *p*-value is less than the threshold of 0.05, it indicates that the laser processing distance has a significant effect on the shear strength of cross-wavy joints.

### 3.3. Interface Analysis

**Figure 6:**
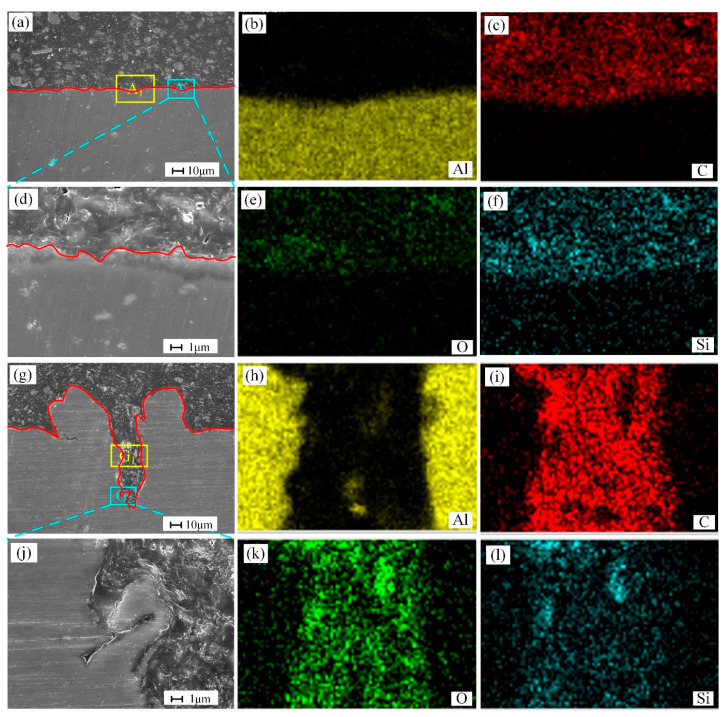
Morphology and element distribution of the adhesive interface (**a**) unengraved joint; (**b**–**f**) distribution of elements in region A_1_; (**d**) enlarged view of the A_2_ region; (**g**) laser engraved joint; (**h**,**i**,**k**,**l**) distribution of elements in region G_1_; (**j**) enlarged view of the G_2_ region.

To know the influence of surface micropattern on bonding strength, the micromorphology of the joint interface was observed before the shear test. Figure 6 shows the interface morphology of the epoxy-aluminum alloy joint before and after laser processing. Figure 6a shows the joint interface before the substrate is engraved. The interface shows a relatively flat contact surface, indicating that the effective bonding area is a relatively flat contact surface. Moreover, it can be observed that the quality of the bonding interface is good, and no obvious cracks and bubbles appear, as shown in Figure 6d,g, which shows the joint interface after laser processing, and the cross-section shows the shape of a goat’s horn. This is due to the partial melting of the material by the laser during the etching process and the subsequent rapid cooling. In addition, the fluted areas formed during laser processing are filled with epoxy adhesive. Compared with the cross-section morphology before laser processing, the effective contact area is significantly increased. Moreover, in Figure 6j, the adhesive and the substrate achieve close contact and defect-free interface bonding.

### 3.4. Fracture Failure Analysis

**Figure 7:**
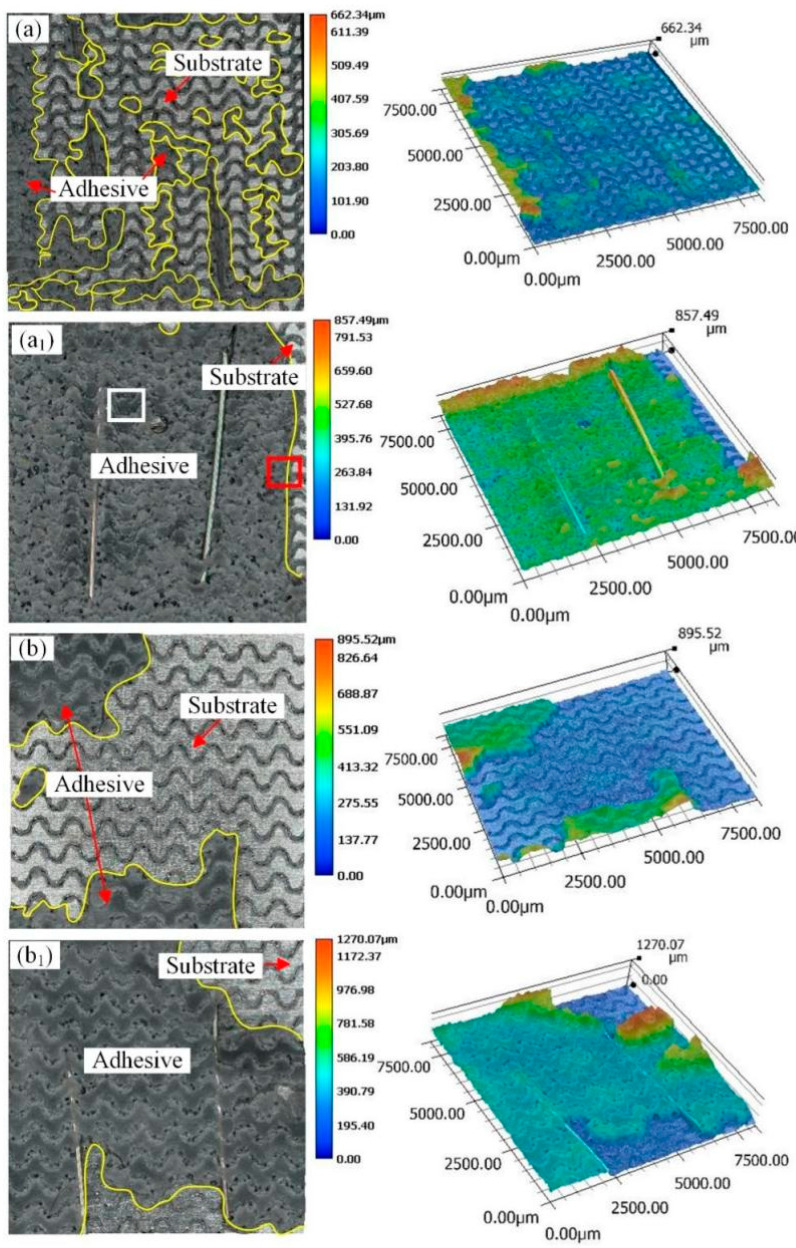
Fracture morphology of parallel wavy micropattern with different laser processing distances (**a**,**a_1_**) sample PW0.5; (**b**,**b_1_**) sample PW0.75.

**Figure 8:**
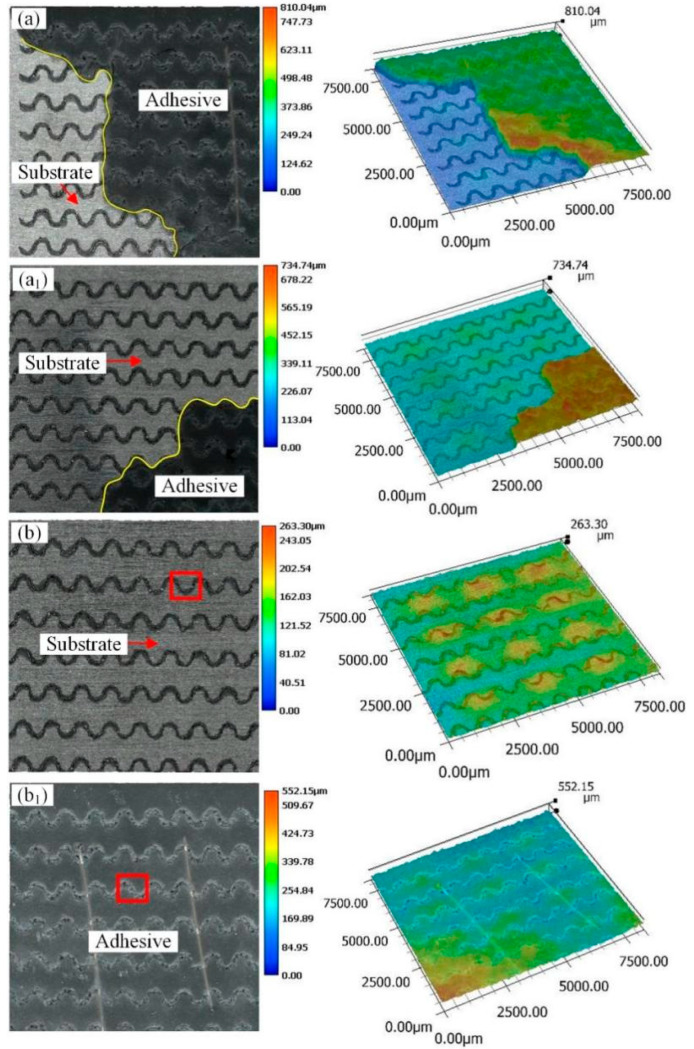
Fracture morphology of parallel wavy micropattern with different laser processing distances (**a**,**a_1_**) sample PW1; (**b**,**b_1_**) sample PW1.25.

Figure 7 and Figure 8 show the macroscopic fracture morphology and three-dimensional morphology of the aluminum alloy bonded joint with parallel wavy micropattern after a pure shear test. The yellow lines in Figure 7 are all adhesives. The fracture morphology of sample PW0.5 is shown in Figure 7a,a_1_. There are adhesive residues on both bonding surfaces, and there are more adhesive residues. This means that most of the cracks appear in the middle of the rubber layer. It shows that the bonding strength between the adhesive and the aluminum substrate with a micropattern is greater than the cohesion of the adhesive itself, and this situation belongs to cohesion failure. But at the same time, a small part of the aluminum alloy substrate can be seen, and this situation appears in the relatively flat place outside the groove. This indicates that there is an adhesive failure in this area. It means that the adhesion of the epoxy adhesive to the unengraved aluminum alloy area is less than the cohesion of the adhesive itself. Therefore, the failure mode of sample PW0.5 belongs to the mixed failure, which is mainly based on the cohesive failure. In combination with Figure 5, it can be found that the shear strength value of PW0.5 in this sample is the largest. The fracture morphology of sample PW0.75 is shown in Figure 7b,b_1_. In this sample, it can also be observed that the adhesive residue is on the two bonding surfaces. However, compared with PW0.5, the cohesive failure ratio of sample PW0.75 is relatively small, resulting in lower bonding strength than sample PW0.5. The failure mode of sample PW0.75 also exists in the form of mixed failure. The fracture morphology of samples PW1 and PW1.25 is shown in Figure 8a,a_1_,b,b_1_. The failure modes of the two samples are mainly adhesive failure. The difference is that the adhesive layer in sample S1 falls off from the two bonding surfaces. Compared with the case of sample PW1.25 falling off from a bonding surface, the fracture of sample PW1 needs to overcome more adhesive force, which results in the shear strength of PW1 being superior to that of PW1.25. It can be seen that, with the increase in laser processing distance, the bonding strength becomes worse and worse. The damage to the adhesive layer is smaller and smaller, the proportion of cohesive failure is gradually reduced, and the adhesive failure is gradually increased.

**Figure 9:**
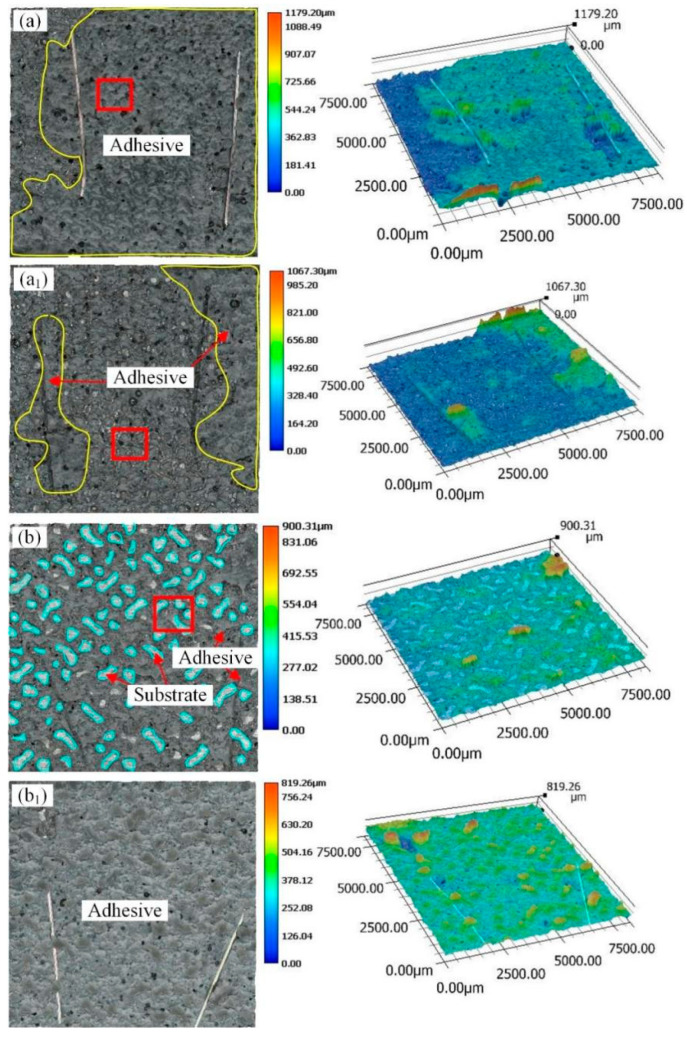
Fracture morphology of cross wave micropattern joints with different laser processing distances (**a**,**a_1_**) sample CW0.5; (**b**,**b_1_**) sample CW0.75.

**Figure 10:**
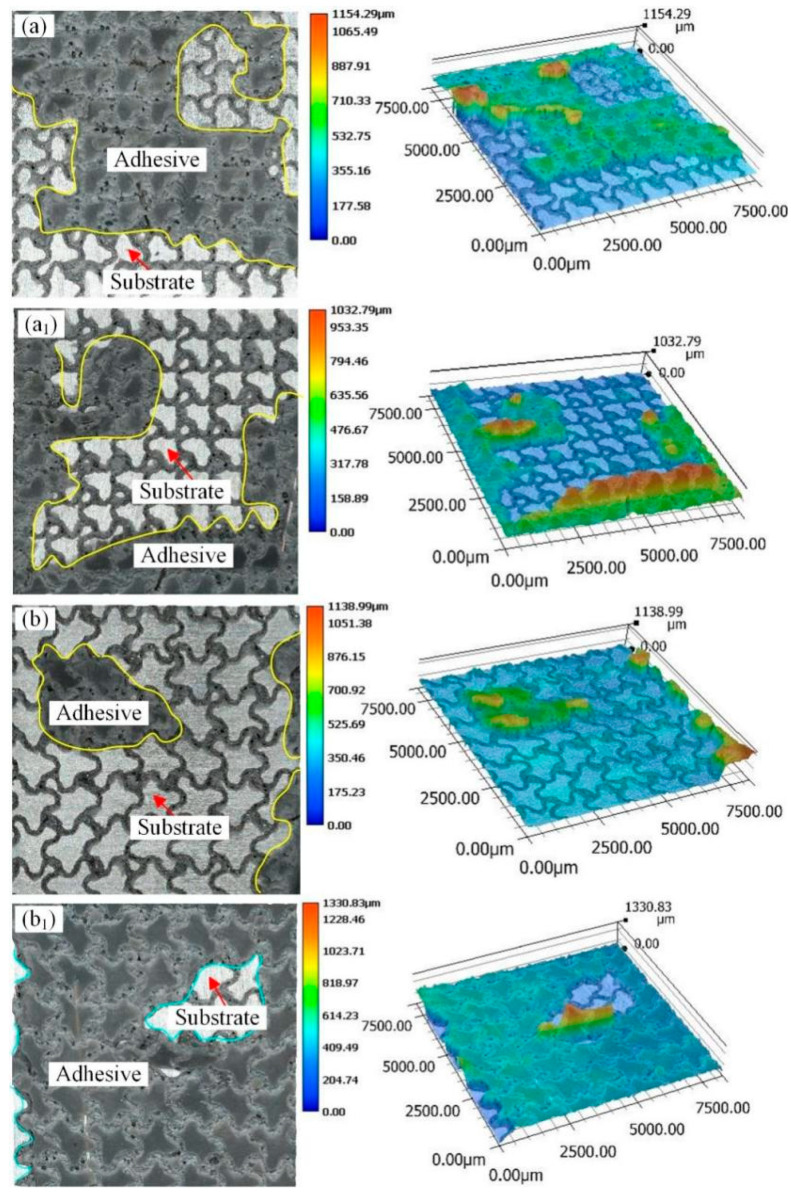
Fracture morphology of cross wave micropattern joints with different laser processing distances (**a**,**a_1_**) sample CW1; (**b**,**b_1_**) sample CW1.25.

Figure 9 and Figure 10 show the macroscopic fracture morphology and three-dimensional morphology of aluminum alloy bonded joints with a cross-wave micropattern after a pure shear test. Among them, the parts circled by the cyan lines are aluminum alloy substrates, and the yellow line area is still an adhesive. In the fracture morphology of sample CW0.5, the rest parts except the yellow lines are mixed failures. There is adhesive residue only in the groove part, and the flat area is an aluminum alloy substrate, as shown in Figure 9a,a_1_. Therefore, the sample CW0.5 is a mixed failure mainly based on cohesive failure. For the sample CW0.75, the mixed failure is also dominated by cohesive failure, as shown in Figure 9b,b_1_. However, in combination with Figure 5, it can be seen that the sample CW0.75 has the largest bonding strength. This is because when the laser sculpts the substrate surface, a portion of the recast material covers the laser path boundary, which further deepens the groove. For the cross-wave micropattern, the smaller the groove distance, the denser the groove pattern will be. These accumulated residues may reduce the coating and penetration of the adhesive. It is difficult for the adhesive to fully fill the gap between the micropattern, thus weakening the bonding strength. For sample CW0.75, the appropriate laser processing distance increases the shear strength of the joint. For samples CW1 and CW1.25, the adhesive layer is completely detached from the two adhesive surfaces, which belongs to the mixed failure mainly caused by adhesive failure. The adhesive residue was found in the grooves, as shown in Figure 10a,a_1_,b,b_1_. Compared with the CW1.25 sample, the laser processing distance of the CW1 sample is relatively smaller and the groove area is bigger. This provides more contact area for adhesive adhesion.

**Figure 11:**
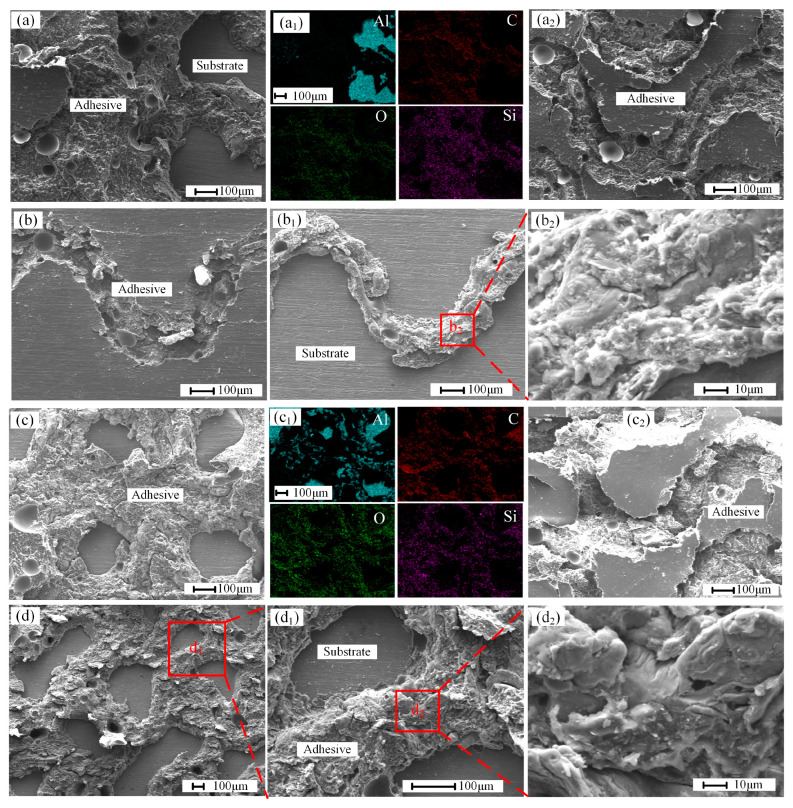
Micromorphology of fracture (**a**–**a_2_**) sample L0.5; (**b**–**b_2_**) sample L1.25; (**c**–**c_2_**) sample CW0.5; (**d**–**d_2_**) sample CW0.75.

In order to further analyze the advantages of the surface micropattern of aluminum alloy in improving the bonding strength, a scanning electron microscope (SEM) was used to observe the damaged sample. Figure 11 shows the microscopic morphology of typical failure samples. Figure 11a,a_1_ is the enlarged area in the red box shown in Figure 7a_1_. Through the element analysis of this area by way of face scanning, it can be observed that the aluminum element is mainly distributed in the flat place, the rest are adhesives, and the grooves are filled with epoxy adhesive. This provides the adhesive with a larger contact area and more mechanical interlocking units, thereby improving bond strength. Figure 11a_2_ is the enlarged area in the white box of Figure 7a_1_. All the areas shown are adhesive layers, and it can be found that some areas of the adhesive layer have cohesive fractures. The fracture part shows the micropattern shape of the other bonding surface. This means that the presence of the substrate surface micropattern further increases the bonding force between the adhesive and the substrate. This is also the case in Figure 11b. It is observed in Figure 11b_1_,b_2_ that the groove of the other bonding surface is also filled with adhesive. These phenomena exist in every sample. Among them, Figure 11b is the enlarged Fig. in the red box of Figure 8b_1_, and Figure 11b_1_ is the enlarged Fig. in the red box of Figure 8b. For the cross micropattern, the fractures of samples CW0.5 and CW0.75 are further analyzed. Figure 11c,c_1_ is the enlarged Fig. in the box of Figure 9a_1_. Figure 11c_2_ is the enlarged figure in the box of Figure 9a. Figure 11d–d_2_ is the enlarged figure in the box of Figure 9b. These can also prove the formation of an effective mechanical lock between the surface micropattern of the substrate and the epoxy adhesive. Thus, the bonding strength of epoxy adhesive and aluminum alloy is enhanced.

**Figure 12:**
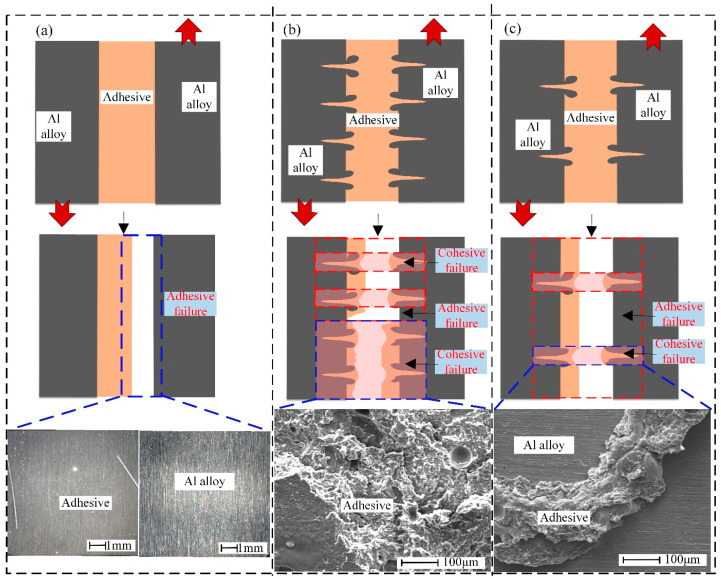
Joint failure mode (**a**) adhesive failure; (**b**) mixed failure predominantly cohesive failure; (**c**) mixed failure predominantly adhesive failure.

The main fracture forms of the joints are summarized in Figure 12. The adhesive layer is completely detached from one of the bonding surfaces when the aluminum alloy substrate is not laser processed. The fracture of the joint presents a complete adhesive failure mode, as shown in Figure 12a. Combined with Figure 5, it can be seen that the epoxy adhesive has the worst shear force with the unengraved aluminum alloy substrate. After laser processing, when the laser processing distance is small (e.g., samples CW0.5, CW0.75), the number of grooves in the same laser processing area is greater, which provides more adhesion area for the adhesive. In combination with Figure 11c,d, it can be seen that the cohesion failure occurs in the grooves. Cohesion failure also occurs between some grooves, as shown in Figure 12b. This is due to the fact that the closer adjacent groove sidewalls also provide more bonding area for the adhesive, with which it forms a mechanical interlock. When the laser processing distance is large (e.g., sample PW1.25), the adhesive layer cohesion failure occurs only within the grooves, and the adhesive failure occurs in the unengraved area outside the groove, as shown in Figure 12c.

**Figure 13:**
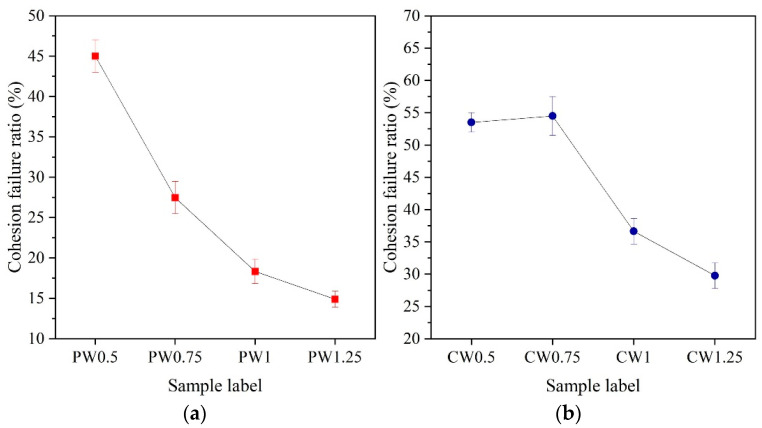
Proportion of cohesive failures (**a**) parallel wavy; (**b**) cross wavy.

Due to the random nature of the breakage of the joint, it was analyzed with the help of VHXComm software 1.2.15.8. The proportion of cohesive failures of parallel rectilinear and crossed wavy shapes at different processing distances can be approximately obtained, and the results are shown in Figure 13. Figure 13a is mainly based on Figure 7 and Figure 8; Figure 13b is mainly based on Figure 9 and Figure 10. In the parallel wave, the proportion of cohesive failure gradually decreases with the increase in laser processing distance. In the cross-wave shape, the proportion of cohesive failure increases first and then decreases with the increase in laser processing distance. The proportion of cohesive failure reflects the bonding state at the interface between the adhesive body and the substrate. Combined with Figure 5, it can be seen that the larger the proportion of cohesive failure, the more conducive and greater the interface strength between the adhesive and the substrate.

## 4. Conclusions

In this study, the effect of laser surface micropattern on the shear properties of adhesive-bonded joints was studied. The influence of different laser processing distances on the shear strength of the bonded joint was investigated. The results of the research are as follows:

(1) The wettability of the 2A12 aluminum alloy substrate surface was significantly improved by laser treatment of a surface micropattern. The water contact angle of the unengraved substrate surface was 84.25° and that of the laser-treated surface (sample PW0.5) was 14.45°. Compared with the unengraved sample, the contact angle is reduced by 83.1%, and the surface of the sample is super hydrophilic.

(2) For the parallel wavy micropattern, the shear strength of bonded joints decreases with the increase in the laser processing distance. When the laser processing distance D = 0.5 mm, the maximum value was 14.04 MPa (sample PW0.5). For the cross-wave micropattern, with the increase in laser processing distance, the adhesive joint first increases and then decreases. When the laser processing distance D = 0.75 mm, the maximum value was 13.74 MPa (sample CW0.75). Compared with the unengraved sample (5.41 MPa), the shear strengths of PW0.5 and CW0.75 were increased by 159.52% and 153.97%, respectively.

(3) The laser processing surface micropattern can effectively increase the contact surface of the adhesive on the substrate. This provides more adhesion points for the adhesive, thereby effectively improving the shear resistance of the joint.

## Figures and Tables

**Table 1:** Chemical composition of 2A12 aluminum alloy.

Composition	Al	Cu	Mg	Mn	Si	Fe	Ti	Zn
wt %	Bal.	3.8–4.9	1.2–2.0	0.4–0.8	≤0.5	≤0.5	≤0.2	≤0.3

**Table 2:** Laser engraving parameters and corresponding sample identification.

Laser Power (W)	Laser Processing Speed (mm/s)	Laser Processing Distance (mm)	Micropatterns Type and Sample Label	
			Parallel Waves	Cross Waves
250	50	0.5	PW0.5	CW0.5
		0.75	PW0.75	CW0.75
		1	PW1	CW1
		1.25	PW1.25	CW1.25

**Table 3:** The measured values of shear strengths for each sample and average values.

Sample Label	Shear Strength of Sample 1 (MPa)	Shear Strength of Sample 2 (MPa)	Average Shear Strength (MPa)
PW0.5	13.98	14.10	14.04
PW0.75	12.04	11.45	11.75
PW1	8.99	10.50	9.75
PW1.25	8.35	8.03	8.19
CW0.5	13.08	12.45	12.77
CW0.75	14.21	13.27	13.74
CW1	10.35	10.72	10.54
CW1.25	9.40	8.99	9.19

**Table 4:** A one-way ANOVA was conducted, as shown in Figure 5b. The specific parameters include the following: the sum of mean squared (*SS*), degrees of freedom (d*f*), mean square error (*MS*), *F*-value, F critical value (*F*_crit_), and *p*-value, and significance evaluation.

Difference Source	*SS*	d*f*	*MS*	*F*	*p*	*F*_crit_	Evaluation
Inter group	269.6729	2	134.84	30.25	0.0001	4.2565	Significance
within group	40.1165	9	4.457				
Total	309.7894	11					

**Table 5:** A one-way ANOVA was conducted, as shown in Figure 5d. The specific parameters include: the sum of mean squared (*SS*), degrees of freedom (d*f*), mean square error (*MS*), *F*-value, F critical value (*F*_crit_), *p*-value, and significance evaluation.

Difference Source	*SS*	d*f*	*MS*	*F*	*p*	*F*_crit_	Evaluation
Inter group	304.704	2	152.352	51.786	0.0000116	4.2565	Significance
within group	26.4778	9	2.942				
Total	331.1818	11					

## Data Availability

The data that support the findings of this study are available from the corresponding author upon reasonable request.
